# Transgenic Analyses of Homer2 Function Within Nucleus Accumbens Subregions in the Regulation of Methamphetamine Reward and Reinforcement in Mice

**DOI:** 10.3389/fpsyt.2020.00011

**Published:** 2020-02-05

**Authors:** Chelsea N. Brown, Elissa K. Fultz, Sami Ferdousian, Sarina Rogers, Elijah Lustig, Ariana Page, John R. Shahin, Daniel M. Flaherty, Georg Von Jonquieres, Camron D. Bryant, Tod E. Kippin, Karen K. Szumlinski

**Affiliations:** ^1^ Department of Psychological and Brain Sciences, University of California, Santa Barbara, Santa Barbara, CA, United States; ^2^ Translational Neuroscience Facility, School of Medical Sciences, University of New South Wales, Sydney, NSW, Australia; ^3^ Laboratory of Addiction Genetics, Departments of Pharmacology and Experimental Therapeutics and Psychiatry, Boston University School of Medicine, Boston, MA, United States; ^4^ Department of Molecular, Cellular and Developmental Biology and the Neuroscience Research Institute, University of California, Santa Barbara, Santa Barbara, CA, United States; ^5^ Center for Collaborative Biotechnology, University of California, Santa Barbara, Santa Barbara, CA, United States

**Keywords:** Homer2, place-preference, self-administration, nucleus accumbens, adeno-associated virus, knock-out

## Abstract

Problems associated with the abuse of amphetamine-type stimulants, including methamphetamine (MA), pose serious health and socioeconomic issues world-wide. While it is well-established that MA’s psychopharmacological effects involve interactions with monoamine neurotransmission, accumulating evidence from animal models implicates dysregulated glutamate in MA addiction vulnerability and use disorder. Recently, we discovered an association between genetic vulnerability to MA-taking and increased expression of the glutamate receptor scaffolding protein Homer2 within both the shell and core subregions of the nucleus accumbens (NAC) and demonstrated a necessary role for Homer2 within the shell subregion in MA reward and reinforcement in mice. This report extends our earlier work by interrogating the functional relevance of Homer2 within the NAC core for the conditioned rewarding and reinforcing properties of MA. C57BL/6J mice with a virus-mediated knockdown of Homer2b expression within the NAC core were first tested for the development and expression of a MA-induced conditioned place-preference/CPP (four pairings of 2 mg/kg MA) and then were trained to self-administer oral MA under operant-conditioning procedures (5–80 mg/L). Homer2b knockdown in the NAC core augmented a MA-CPP and shifted the dose-response function for MA-reinforced responding, above control levels. To determine whether Homer2b within NAC subregions played an active role in regulating MA reward and reinforcement, we characterized the MA phenotype of constitutive *Homer2* knockout (KO) mice and then assayed the effects of virus-mediated overexpression of Homer2b within the NAC shell and core of wild-type and KO mice. In line with the results of NAC core knockdown, *Homer2* deletion potentiated MA-induced CPP, MA-reinforced responding and intake, as well as both cue- and MA-primed reinstatement of MA-seeking following extinction. However, there was no effect of Homer2b overexpression within the NAC core or the shell on the KO phenotype. These data provide new evidence indicating a globally suppressive role for Homer2 in MA-seeking and MA-taking but argue against specific NAC subregions as the neural loci through which Homer2 actively regulates MA addiction-related behaviors.

## Introduction

Amphetamine-type stimulants, including methamphetamine (MA), are the most highly abused psychostimulants in the world, with an estimated 29 million users worldwide in 2017 (1) . Despite the prevalence and severity of MA Use Disorder, the lack of knowledge regarding the neurobiological substrates underlying risk, development and severity impedes therapeutic progress. MA reinforcement and psychomotor activation involves monoamine release and reuptake inhibition, particularly within dopaminergic neurons from the ventral tegmental area (VTA) to the nucleus accumbens (NAC) (2)., Accumulating evidence supports the role of glutamate transmission, especially glutamatergic projections from the prefrontal cortex (PFC) to the NAC, in both MA addiction vulnerability and the long-term neuroplasticity maintaining the MA-addicted state (3–6).

It has been known for decades that binge-like, high-dose (> 4 mg/kg) MA exposure induces glutamate-dependent neurotoxicity within the dorsal striatum (7). However, subchronic administration of subtoxic MA doses (< 2 mg/kg) can also elevate extracellular glutamate within the NAC (3, 8). In addition, such exposure is sufficient to increase the expression/function of mGlu1/5 glutamate receptors and their associated scaffolding protein Homer2 within this region (8). Indeed, a survey of the extant literature on animal models of MA abuse supports a correlative link between potentiated indices of glutamate signaling and addiction-related behavior, including self-administration, MA-induced reinstatement of drug-seeking after abstinence or extinction, incubation of MA-craving, and conditioned place-preference (CPP) (8–17).

Supporting a link between NAC glutamate and MA addiction vulnerability, drug-naïve mice selectively bred for high MA intake (MAHDR) exhibit several glutamate anomalies within the NAC, relative to MALDR mice selectively bred for low MA drinking (18–22). These differences include elevated basal and MA-induced increases in extracellular glutamate, increased expression of Homer2 and mGlu5, and decreased expression of the EAAT3 glutamate transporter responsible for clearing synaptic glutamate (3, 8). Further, NMDA glutamate receptor antagonists attenuate MA-conditioned reward and behavioral sensitization (14), while pharmacological manipulations of extracellular glutamate in the NAC bidirectionally regulate the expression of MA-conditioned reward in B6 mice (8). These results provide causal evidence for a relationship between glutamate and MA-induced behavior. Finally, small hairpin RNA (shRNA)-mediated knockdown of Homer2 expression in the shell subregion of the NAC reduces the magnitude of both a MA CPP and oral MA intake during operant-conditioning procedures (8), indicating for the first time a causal role for Homer2, at least within the NAC shell, in regulating the rewarding and reinforcing properties of MA.

The present study sought to extend our earlier results in the NAC shell (8) to the NAC core subregion and to probe the bidirectionality of the effects of transgenic manipulations of NAC Homer2 expression on MA addiction-related behaviors. The core and shell subregions of the NAC have distinct functions, connectivity, and pharmacology that are still being characterized within the context of addiction (23). Current theories argue that the NAC core is embedded within subcircuits involved in decision-making by signaling the motivational value of expected goals to guide drug-seeking in drug-experienced animals. In contrast, the NAC shell appears to be more involved in the initial affective valence of the drug during early drug experience (23). As Homer2 expression within both subregions is correlated with MA addiction vulnerability in mouse models (8), we first examined the effects of knocking down Homer2 expression in the NAC core on MA-induced CPP and the acquisition of oral MA self-administration in inbred C57BL/6J (B6) mice. The combined results of our knockdown studies suggest opposing roles for Homer2 within the NAC shell and core in regulating MA reward and reinforcement. To determine whether Homer2 contributes to the development of MA CPP and oral intake, we also determined the effects of upregulating Homer2 expression in both NAC subregions on the behavior expressed by constitutive *Homer2* knockout (KO) mice and their wild-type (WT) counterparts.

## Materials and Methods

### Subjects

The knockdown studies employed adult, male C57BL/6J (B6) mice (~8 weeks of age; The Jackson Laboratory, Sacramento, CA). The remaining studies used both male and female adult (6–8 weeks of age) *Homer2* KO and wild-type (WT; on a mixed 129X1/svJ X C57BL/6J background) mice [see (24)] that were bred in-house from the mating of heterozygous breeder pairs in the Psychological and Brain Sciences vivarium at UCSB. Animals were housed in groups of 3–5 mice in standard ventilated polycarbonate cages, under standard, reverse-light, housing conditions in an AAALAC-accredited vivarium (lights on/off: 2200/1000 h), with *ad libitum* access to food and water. All behavioral procedures were conducted during the dark phase of the circadian cycle. All procedures were consistent with NIH guidelines and approved by the Institutional Animal Care and Use Committee of UCSB.

### General Experimental Design

**Figure 1:**
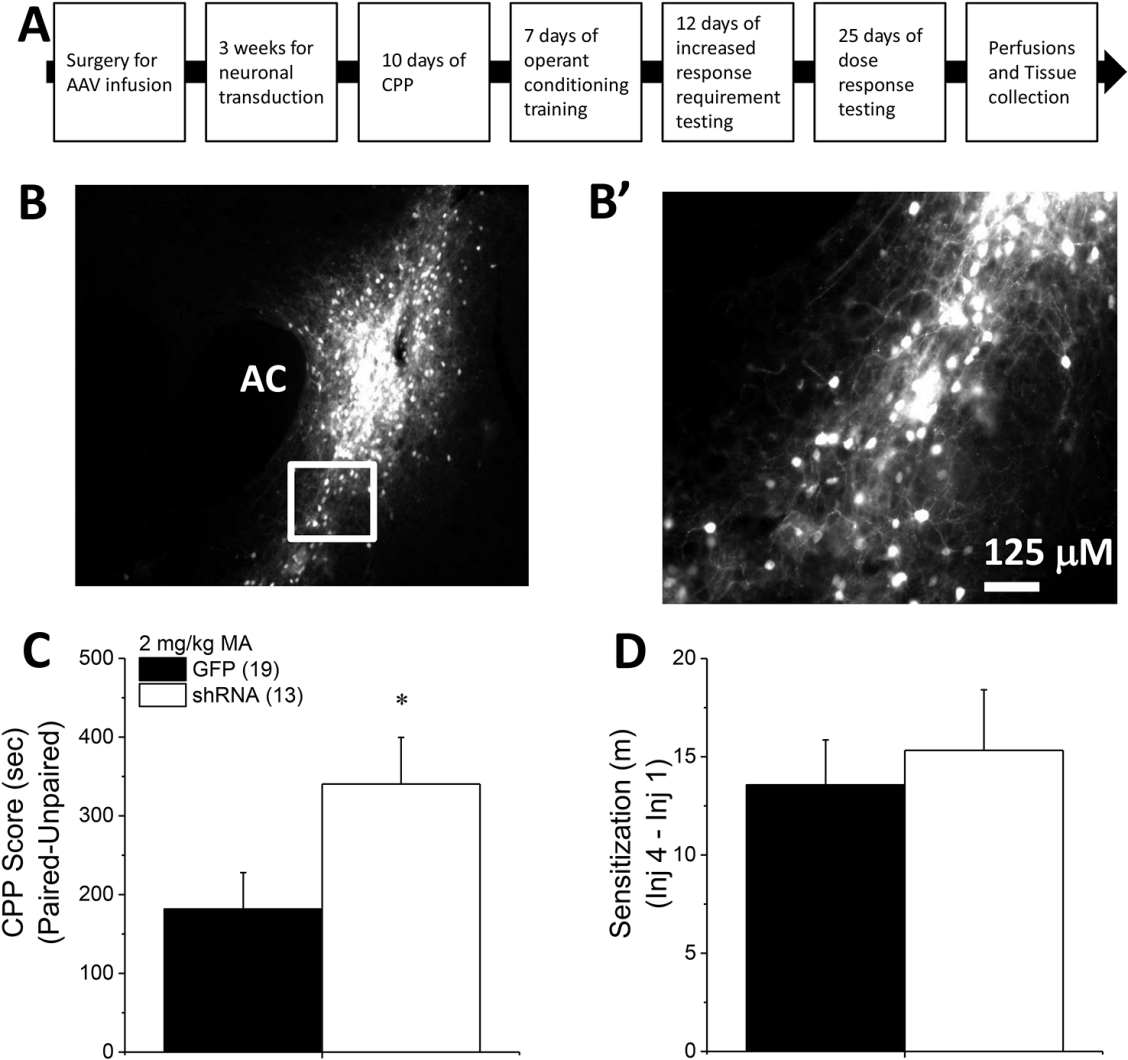
Homer2b knockdown in the nucleus accumbens (NAC) core potentiates a methamphetamine (MA)-induced CPP. **(A)** The procedural timeline for the study examining the effects of shRNA-mediated knock-down of Homer2b within the NAC core. Representative micrographs of the neuronal transduction within the NAC core by green fluorescent protein (GFP)-tagged adeno-associated viral vector (AAV)-shRNA against Homer2b at 10 X magnification **(B)** and 40 X magnification **(B’)**. AC, anterior commissure. **(C)** shRNA infusion potentiated MA-induced place conditioning, without altering the magnitude of locomotor sensitization that developed during conditioning **(D)**. The data represent the means ± SEMs of the number of mice indicated in Panel **B**. ^*^p < 0.05 vs. GFP.

Homer2 within the NAC regulates both cocaine- (25) and alcohol-induced (26–30) changes in behavior in murine models, but the subregional specificity of Homer2’s role in MA-related behavior has received relatively little experimental attention (8). Thus, two experiments were conducted to further address the role for NAC Homer2 expression in gating the rewarding and reinforcing properties of MA. The first experiment in this report sought to extend the results of a prior study of the NAC shell (8) to the NAC core by determining whether or not Homer2 expression within the NAC core is necessary for MA reward/reinforcement. To accomplish this, the first experiment in this report employed a similar experimental design and approach as that described in our previous report (8), which involved knocking down Homer2b expression in the NAC core of B6 mice using an adeno-associated viral vector (AAV) carrying a small hairpin RNA (shRNA) against *Homer2b*. Control animals were infused with an AAV carrying green fluorescent protein (GFP). The details of the AAV-shRNA construct and the control AAV are provided in Klugmann and Szumlinski (31) and Cozzoli etal. (29) and the details of the specific procedures employed in this shRNA study are provided in the subsections below. A time-line of the procedures is provided in **Figure 1A**.

Combined, the results of our prior shRNA study of the NAC shell (8) and those of the present study of the NAC core (see Results below) argued that Homer2 expression within the NAC shell and core plays opposing roles in gating MA reward/reinforcement, with Homer2 in the shell promoting, and Homer2 in the core, suppressing MA addiction-related behaviors. Thus, a follow-up experiment was conducted to determine whether or not mimicking a MA-induced increase in Homer2 expression within the NAC shell and core (8) would be sufficient to respectively promote and suppress MA-induced place- and operant-conditioning. To address this question, we employed an AAV *Homer2b*-cDNA strategy similar to that used in previous studies from our laboratory (25, 26, 32). As in our earlier work [e.g., (25)], we infused a *Homer2b* AAV-cDNA construct [see (25) and (31) for details of the cDNA construct] into the NAC shell or core of *Homer2* WT and constitutive KO mice, the latter of which enabled determination of an active role for Homer2 within each subregion in gating behavior. As the effects of constitutive *Homer2* deletion upon MA addiction-related behaviors had yet to be characterized, we first compared the MA place- and operant-conditioning phenotypes of *Homer2* KO and WT mice on a mixed B6-129 hybrid genetic background. Then, we replicated the experiment in a second cohort of *Homer2* KO and WT mice infused with either the AAV-cDNA or -GFP control. A time-line of procedures is presented in **Figure 5A**.

### Surgeries and AAV Infusion

The surgical procedures to infuse the AAVs carrying either shRNA-Homer2b, cDNA-Homer2b, or cDNA-GFP were consistent with those previously described by our laboratory (8, 29, 33). For B6 mice, we used the following stereotaxic coordinates from Bregma (in mm): for core, AP: +1.3; ML: ± 1; DV: −4.3; for shell, AP: +1.3; ML: ± 0.5; DV: −4.8. Based on our experience conducting craniotomies on B6-129 hybrid mice [e.g., (25, 29, 33)], the following stereotaxic coordinates were used for *Homer2* KO and WT mice: for core, AP: +1.4; ML: ± 1; DV: −4.3; for shell, AP: +1.4; ML: ± 0.5; DV: −4.6. Mice were anesthetized with 1.5% isoflurane and positioned on the stereotaxic apparatus. Thirty gauge microinjectors (12 mm) were lowered bilaterally, directly into the core or shell. AAVs were infused at a rate of 0.10 µl/min for 5 min (total volume/side = 0.50 µl), and injectors were left in place for an additional 5 min prior to closing the incision site with tissue adhesive. The shRNA and cDNA infusions procedures have been demonstrated previously to reduce and increase, respectively, Homer2b protein expression in mouse brain by approximately 50% (31, 33, 34). Animals were left in their home cages for a minimum of 3 weeks prior to behavioral testing to allow for maximal neuronal transduction (31).

### Place-Conditioning and Locomotor Activity

MA place-conditioning procedures also followed those previously employed by our laboratory (8) and included three main phases: habituation/preconditioning test (day 1), MA/saline (SAL) conditioning (days 2–9), and a postconditioning test (day 10, post-test). The apparatus consisted of two distinct compartments—one with black and white marble-patterned walls and a textured floor, and the other with wood-patterned walls and a smooth Plexiglas floor. During the habituation and post-test sessions, mice were allowed free-access to both compartments for 15 min *via* a divider with a door. During conditioning, mice received 2 mg/kg MA intraperitoneal (IP) injections and were immediately confined to one of the compartments. On alternating days, mice were injected with an equivalent volume of SAL (10 ml/kg) and confined to the other compartment. Each conditioning session was 15 min in duration and mice received four conditioning sessions for each unconditioned stimulus. Overall, mice did not exhibit a strong preference for one compartment vs. the other during the habituation session, so the time spent on the SAL-paired side during the post-test was subtracted from the time spent on the MA-paired side to calculate a CPP score (8, 35). This CPP score served to index the direction and magnitude of the MA-conditioned reward. During each 15-min session, the locomotor activity of the animals was recorded by digital video cameras, interfaced with a PC-type computer equipped with ANY-Maze software (Stoelting), recorded the distance traveled (in m) during each of the sessions. As in our prior studies [e.g., (8)], MA-induced locomotor sensitization was measured by subtracting the distance travelled during the first 15-min MA-conditioning session from that on the fourth/last MA-conditioning session.

### Operant-Conditioning

In our prior study of the effects of Hoemr2 knock-down in the NAC shell (8), the generalization of a place-conditioning phenotype to operant-conditioning for MA reinforcement was determined using a within-subjects design. To the best of our knowledge, a parametric analysis of the effects of prior behaviorally non-contingent MA upon subsequent drug-taking has not been performed. Thus, we cannot speak to any potential effects our place-conditioning procedures might have upon the MA self-administration of the mice. However, we do know from our prior study of B6 mice, that a mere history of non-contingent MA treatment (four injections of 2 mg/kg MA, as employed in the present study) does not necessarily promote subsequent MA reinforcement/intake as MA-injected mice self-segregate into high versus low MA-taking phenotypes when allowed to orally self-administer the drug (8). To be consistent with our prior study (8), following place-conditioning procedures, mice were trained in daily 1-h sessions to nose-poke for delivery of unadulterated MA solutions (prepared in tap water; reinforcer volume = 20 µl). Standard mouse operant-conditioning chambers (MedAssociates, St Albans, VT, USA) were used to measure instrumental responding for MA. Operant chambers were fitted with two nose-poke holes, with a liquid receptacle located in-between and chambers were housed in ventilated, sound-attenuated chambers. Responses in the active (MA-associated) hole resulted in the activation of the infusion pump, delivery of 20 µl MA into the receptacle, and the presentation of a 20-s light/tone compound stimulus. During the 20-s MA-delivery period, further responding in the active hole was recorded but had no programmed consequences. Throughout the session, responding in the inactive hole had no programmed consequences but was recorded to assess the selectivity of responding in order to determine reinforcer efficacy. Mice were first trained for 7 days to nose-poke for delivery of a 10-mg/L MA solution under an FR1 schedule of reinforcement. Animals that did not reach the acquisition criteria of at least 10 active nose-pokes during the 1-h session, with greater than 65% of their total nose-pokes directed at the active hole were excluded from the study. Using these criteria, 9/48 mice were excluded from the shRNA study and 20/114 mice were excluded from the cDNA studies. As in our prior study of the NAC shell (8), we next progressively increased the number of nose-pokes required for delivery of the 10 mg/L MA reinforcer (maintaining the 20-s time-out) over subsequent days (4–5 days/schedule). We then conducted a dose-response study of MA reinforcement and intake (5–80 mg/L) under the initial FR1 (20-s time-out) reinforcement schedule (5 days/dose) as data indicated an inverse relationship between MA intake and reinforcement schedule (see Results). Given the inverse relationship between operant-responding and reinforcement schedule, we opted to forego this phase of testing in the cDNA study and animals proceeded from training directly into dose-response testing. In the operant-conditioning study of *Homer2* WT and KO mice, technical issues interfered with the testing of 13 of the 21 WT mice at the 80 mg/L concentration. As such, the data from this concentration were analyzed separately from the rest of the dose-response function.

At the end of each 1-h operant session, the volume of solution remaining in the receptacle was determined by pipetting. Mice were returned to the colony room and left undisturbed until the next day. Total MA intake was calculated each day by subtracting the volume of MA remaining in the receptacle from the total volume delivered to determine the total volume of MA consumed. The volume consumed was converted into mg consumed based on the concentration of the solution and then amount of MA intake was expressed as a function of body weight (in mg/kg), which was measured weekly (8).

### Extinction and Reinstatement of the Operant Response

In the cDNA study, the strength of the conditioned operant response was established by repeatedly testing mice in daily operant sessions in a MA-free state, with no light/tone stimulus, until the number of active nose-pokes in a 1-hr session dropped to 25% of initial MA-free responding (i.e., extinction). Animals that did not reach these extinction criteria within 30 days were excluded from the remainder of the study. Two additional mice from the cDNA studies were excluded for failing to reach extinction criteria. This extinction procedure was conducted immediately upon the completion of dose-response testing (see above). Following extinction, AAV-infused mice were then subjected to a series of reinstatement of MA-seeking tests in which responding in the active hole resulted in the presentation of only the light/tone stimulus previously predictive of MA delivery (i.e., MA reinforcement was withheld during reinstatement testing). For reinstatement testing, mice were administered a once-daily IP injection of 0.0 (SAL), 0.5 or 0.25 mg/kg MA, with doses increased across days, to examine the degree of cue- and MA-induced reinstatement of the conditioned response. Immediately following injection, mice were placed into the operant-conditioning chamber for a period of 1 h, at which time they were removed and returned to their home cages and the number of active versus inactive nose-pokes were recorded.

### Histology

The goal of this study was to determine the subregional specificity of the effects of AAV-mediated Homer2 manipulations within the NAC for MA addiction-related behavior. As such, we deemed it important to determine the neuroanatomical specificity of AAV infusion and thus, employed immunohistochemical, *in lieu* of immunoblotting, procedures to localize neuronal transduction within the NAC shell versus core. For this, animals were euthanized with an overdose of Euthasol (Virbac AH, Fort Worth, TX, USA) and transcardially perfused with phosphate-buffered saline (PBS), followed by 4% paraformaldehyde. Brains were then removed and cold-stored in PBS until slicing. Tissue was sectioned (40 µm) along the coronal plane on a vibratome at the level of the NAC. As in our recent work (8), localization of the transfection of neurons by our shRNA-Homer2b, as well as by our GFP control viruses, was examined using an anti-GFP antibody (Invitrogen, Carlsbad, CA, USA; 1:200 dilution) and fluorescence microscopy. As in our prior work (8, 25, 26), tissue from cDNA-Homer2b infused mice was stained with a mouse antihemagglutinin (HA) primary antibody (Biolegend, San Diego, CA, USA; 1:1,000 dilution) to visualize the viral construct, followed by a biotinylated antimouse secondary IgG (Vector Laboratories, Burlingame, CA, USA; 1:2,000 dilution), and visualized with 3,3’-diaminobenzidine (DAB). Poststaining, all tissue was mounted on slides and cover-slipped. Slides were viewed using a Nikon Eclipse E800 microscope equipped with a Hamamatsu CCD camera (model C4742-95) and MetaMorph imaging software (Molecular Devices, Sunnyvale, CA, USA). Only mice exhibiting localized neuronal transduction within the NAC shell and core subregions were included in the statistical analyses of the results.

### Statistical Approaches

The effects of Homer2b knockdown in the NAC core upon place-conditioning related measures were analyzed using t-tests. The operant-conditioning data were analyzed using multivariate ANOVAs, with the between subjects factors of Sex and AAV (GFP vs. shRNA or GFP vs. cDNA) and/or Genotype (WT vs. *Homer2* KO) and the within-subjects factors of Day, FR schedule, and Dose, when appropriate. As initial analyses of the data for both place- and operant-conditioning in *Homer2* WT and KO mice indicated no main Sex effects or interactions, the data were collapsed across sex prior to reanalyses. As described above, the data for *Homer2* WT/KO mice tested for the self-administration of 80 mg/L MA were analyzed separately using t-tests. Two-tailed Pearson correlational analyses were also conducted to relate dependent measures with CPP score. α = 0.05 for these analyses. The effects of *Homer2* KO on the dose-response function for MA-induced place-conditioning were analyzed using ANOVAs, with the between-subjects factors of Genotype (WT vs. KO) and Dose (0.5–4.0 mg/kg MA, 4 levels). All data was analyzed using SPSS ver 12 (IBM) and for all ANOVAs, the homogeneity of variance was confirmed. Alpha was set at 0.05 for all analyses.

## Results

### *Homer2* Knockdown in the NAC Core Augments a MA CPP in B6 Mice

To extend recent results for the NAC shell (8) to the core subregion, B6 mice were infused with an AAV carrying shRNA to knockdown *Homer2b* in the NAC core and then tested for MA-induced CPP. Expression of the AAV was confirmed as confined to the NAC core using fluorescence microscopy (**Figures 1B, B’**). shRNA-infused mice exhibited higher CPP following four pairings of 2 mg/kg MA than GFP-infused controls (**Figure 1C**) [t(30) = 2.14, p = 0.04]. The shRNA-Homer2b NAC core infusion did not affect the acute locomotor response to MA [data not shown; t(30) = 0.39, p = 0.70], nor did it alter the magnitude of MA-induced locomotor sensitization that developed over the course of the conditioning (**Figure 1D**) [t(30) = 0.47, p = 0.64]. These data indicate that Homer2 within the NAC core normally suppresses the positive affective and motivational valence of MA, independent of effects upon without interfering with the locomotor-activating effects of the drug.

### *Homer2* Knockdown in the NAC Core Augments Oral MA Reinforcement Intake in B6 Mice

**Figure 2:**
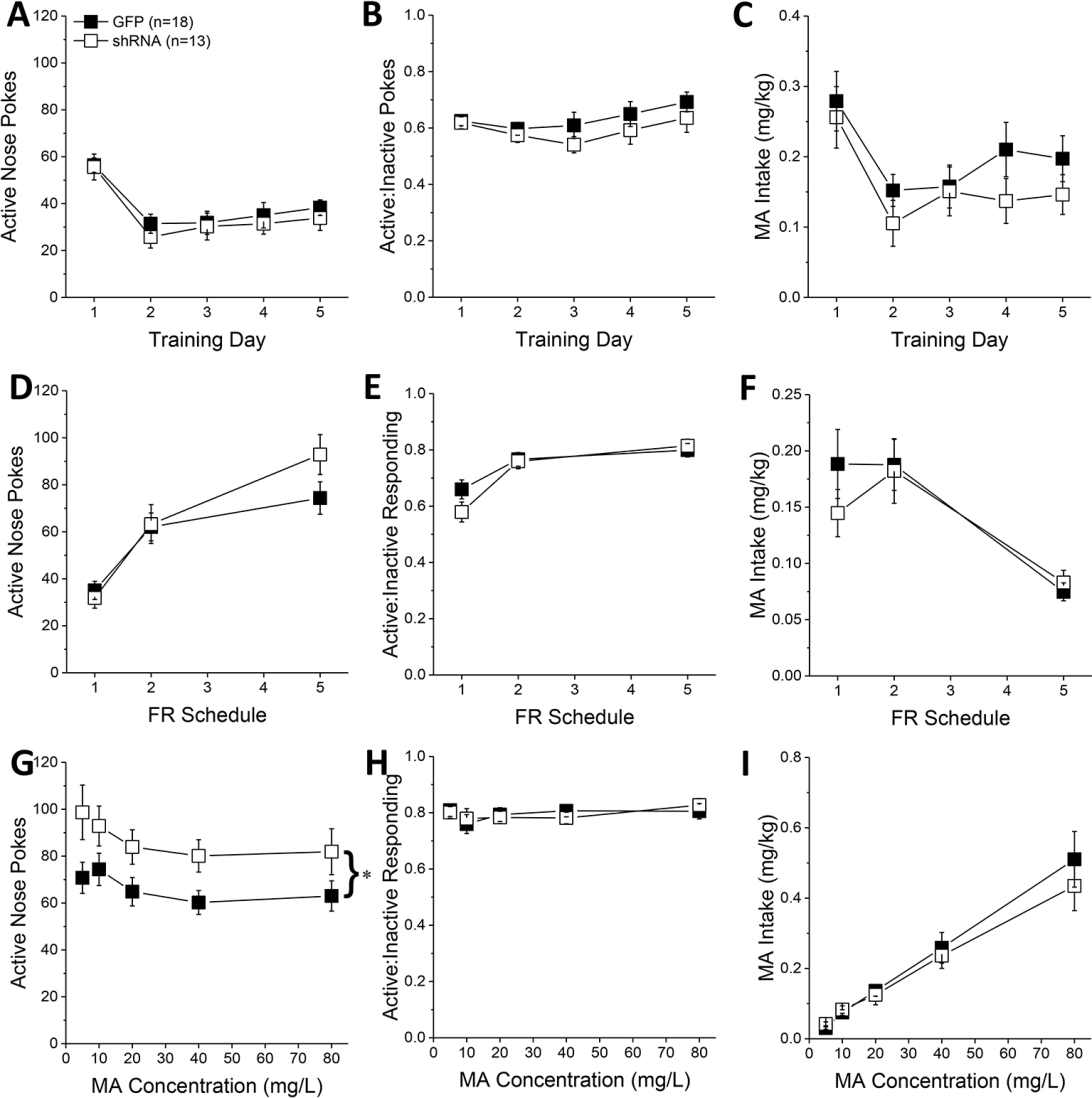
Homer2b knockdown in the nucleus accumbens (NAC) core increases methamphetamine’s (MA’s) reinforcing efficacy without altering MA intake. shRNA against Homer2 did not influence: **(A)** the number of active nose pokes, **(B)** the relative responding on the active versus inactive hole or **(C)** MA intake during the first 5 days of self-administration training (10 mg/L MA as reinforcer). **(D–F)** shRNA infusion also did not alter these measures when mice were tested under increasing response requirements on an FR schedule of reinforcement. **(G)** shRNA infusion shifted the dose-response function for active hole poking upwards of green fluorescent protein (GFP) controls but did not affect the dose-response functions for **(H)** response allocation or **(I)** MA intake. The data represent the means ± SEMs of the number of mice indicated in Panel **A**. ^*^p < 0.05 vs. GFP [main adeno-associated viral vector (AAV) effect].

During the first 5 days of self-administration training under an FR1 reinforcement schedule, both GFP and shRNA animals exhibited a similar pattern of active nose-pokes (**Figure 2A**) [Day effect: F(1,29) = 28.33, p < 0.0001; AAV effect, interaction: p’s > 0.10], ratio of active vs. inactive responding (**Figure 2B**) [Day effect: F(1,29) = 2.56, p = 0.04; AAV effect and interaction, p’s > 0.20], and MA intake (**Figure 2C**) [Day effect: F(1, 29) = 12.20, p = 0.002; AAV effect, interaction: p’s > 0.10]. When tested under increasing response requirements, responding on the active lever increased (**Figure 2D**) [FR effect: F(1,29) = 76.00, p < 0.0001], the ratio of active vs. inactive responding increased (**Figure 2E**) [FR effect: F(1,29) = 25.32, p < 0.0001], and MA intake decreased (**Figure 2F**) [FR effect: F(1,29) = 20.17, p < 0.0001], but there was no effect of Homer2b knockdown on any of these measures (**Figures 2D–F**; AAV effects and interactions, all p’s > 0.20). Thus, Homer2 within the NAC core is not necessary for the acquisition of oral MA self-administration or MA demand, at least when behavior is reinforced by a low, 10 mg/L MA concentration.

In contrast, Homer2b knockdown in the NAC core shifted upwards the dose-response function for active nose-poking behavior (**Figure 2G**) [AAV effect: F(1,29) = 5.31, p = 0.03; Dose effect: F(1,29) = 4.65, p = 0.002; interaction, p = 0.799], without impacting the ratio of active vs. inactive responding (**Figure 2H**; AAV X Dose ANOVA, p’s > 0.35), or the dose-response function for MA intake (**Figure 2I**) [Dose effect: F(1,29) = 77.35, p < 0.0001; AAV effect, interaction, p’s > 0.30]. These data indicate that Homer2 within the NAC core normally curbs the reinforcing efficacy of MA in mice with a history of self-administration, but this effect does not translate into a change in MA intake.

### Constitutive *Homer2* KO Increases Ma-Induced CPP

**Figure 3:**
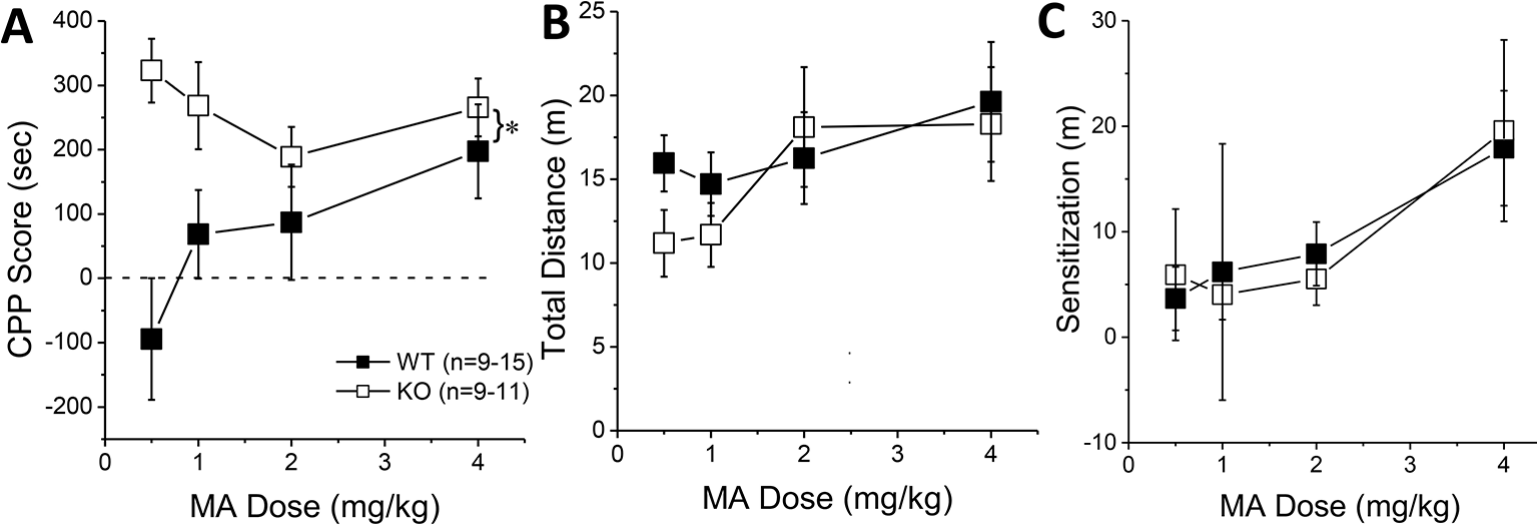
Constitutive *Homer2* deletion augments a methamphetamine (MA)-induced conditioned place-preference (CPP). When compared to wild-type (WT) mice, *Homer2* knockout (KO) animals exhibited **(A)** a shift upwards in the dose-response function for a MA-induced CPP. In contrast, gene deletion did not alter the dose-response functions for **(B)** acute MA-induced locomotor activity or **(C)** the increase in locomotor activity from the first to the last MA-conditioning session (sensitization). The data represent the means ± SEMs of the number of mice indicated in Panel **A**. ^*^p < 0.05 vs. WT (main Genotype effect).

The results of our shRNA study above indicate that Homer2 within the NAC core normally suppresses behavioral indices of MA reward and reinforcement, which is a finding opposite to that reported for Homer2 in the NAC shell (8). Thus, we employed a complementary AAV-cDNA strategy (25, 26, 29) in WT littermates and *Homer2* KO mice to determine whether Homer2 in the NAC shell promotes, while that in the core suppresses, MA place- and operant-conditioning. We know that *Homer2* KO mice exhibit greater sensitivity to the psychomotor-activating effects of MA (36); however, their MA reward/reinforcement phenotype has yet to be characterized. Therefore, we first assayed the effects of a constitutive *Homer2* KO on MA place- and operant-conditioning. A genotypic comparison of the dose-response function for the time spent in the MA-paired vs. -unpaired side during the post-test phase of place-conditioning indicated greater MA-induced CPP, irrespective of MA dose (**Figure 3A**) [Genotype effect: F(1, 86) = 14.83, p < 0.0001; Genotype X Dose: F(3, 86) = 2.54, p = 0.06], although the genotypic difference in MA-conditioned behavior was most obvious at lower MA concentrations. Despite exhibiting potentiated MA-conditioned reward, *Homer2* KO mice did not differ significantly from WT littermate controls regarding the acute locomotor stimulatory effects of MA during the first conditioning session (**Figure 3B**) (Genotype X Dose ANOVA, all p’s > 0.14) or in the capacity of the four MA injections to elicit a dose-dependent sensitization of locomotion during the conditioning phase of the study, as determined by the difference in the distance traveled from the first to the forth injection (**Figure 3C**) [Dose effect: F(1,79) = 8.00, p < 0.0001; Injection: F(3,237) = 8.94, p < 0.0001; Dose X Injection: F(9,237) = 2.47, p = 0.01; Genotype X Dose: F(3,79) = 2.23, p = 0.09; all other p’s > 0.30]. While this result contradicts our earlier report, these conditioning sessions were only 15-min long, while in Szumlinski et al. (36), the sessions were 1 h so the difference in duration of testing likely mitigated genotypic differences.

### Constitutive *Homer2* KO Increases MA-Reinforcement and Intake

**Figure 4:**
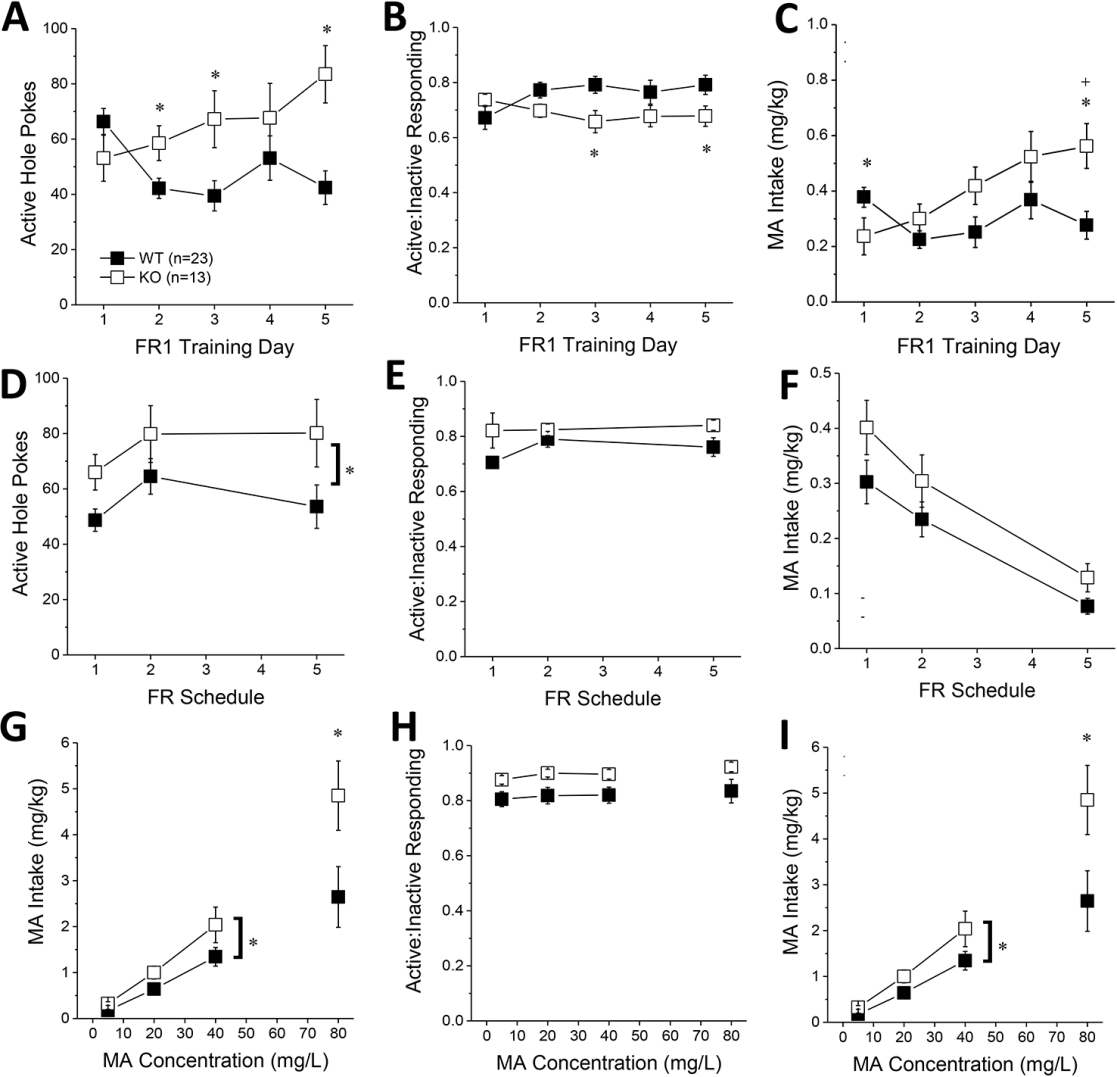
Constitutive Homer2 deletion increases methamphetamine (MA) reinforcement and intake. When compared to WT mice, Homer2 knockout (KO) mice exhibited a greater: **(A)** the number of active nose pokes, **(B)** relative responding on the active versus inactive hole and **(C)** MA intake during the first 5 days of self-administration training (10 mg/L MA as reinforcer). **(D)** *Homer2* KO mice also exhibited more active hole responding under increasing response requirement but did not differ from WT mice regarding **(E)** response allocation or **(F)** MA intake during this phase of testing. Relative to WT mice, the dose-response function for active hole-responding was shifted upwards **(G)**, without a change in that for response allocation in the active hole **(H)**. **(I)** KO mice also consumed more MA than wild-type (WT) mice across the range of doses tested. The data represent the means ± SEMs of the number of mice indicated in Panel **A**. ^*^p < 0.05 vs. WT (main Genotype effect).

The number of active hole pokes emitted by KO mice progressively increased across training days, whereas the responding of WT mice fluctuated during early training (**Figure 4A**) [Genotype X Day: F(4, 140) = 5.71, p < 0.0001]. *Post hoc* analyses indicated greater active hole responding in KO versus WT mice on day 2, 3, and 5 of training (**Figure 4A**; t-tests, p’s < 0.03). No genotypic differences were observed for the number of inactive hole pokes (data not shown; Genotype X Day, all p’s > 0.25). However, KO mice tended to exhibit lower active vs. inactive responding than WT mice during early training (**Figure 4B**) [Genotype X Day, F(4,140) = 3.59, p = 0.008], with *post-tests* indicating significantly lower relative responding on Days 3 and 5 of training (t-tests, p’s < 0.04). Despite the lower active nose-poke responding in KO, only the KO mice escalated their MA intake during early training (**Figure 4C**) [Genotype X Day: F(4, 140) = 5.98, p < 0.0001]. While *Homer2* KOs exhibited significantly lower MA intake than WT animals on the first training day [t(35) = 2.05, p = 0.05], their MA intake was significantly higher than WT animals by the 5^th^ training day [t(35) = 3.18, p = 0.003]. Thus, constitutive *Homer2* deletion increases low-concentration MA reinforcement and intake during early training in a manner similar to shRNA-mediated knockdown of Homer2b expression within the NAC core.

When the response requirement for reinforcement by 10 mg/L MA progressively increased across days, KO mice exhibited more active hole responding, overall, than WT mice (**Figure 4D**) [Genotype effect: F(1,35) = 5.27, p = 0.03; interaction: p > 0.6]. The number of inactive hole pokes declined with increasing response requirement (data not shown) [Schedule effect: F(2,70) = 15.85, p < 0.0001] but was not influenced by genotype (all p’s > 0.60). In contrast to the early acquisition phase (**Figure 4B**), KO mice exhibited slightly higher active hole response allocation than WT mice during this phase of testing (**Figure 4D**) [Genotype effect: F(2,70) = 3.63, p = 0.07; Day effect and interaction, p’s > 0.15]. The MA intake of KO mice was also slightly higher than WT controls as response requirement increased (**Figure 4E**) [Genotype effect: F(1,35) = 3.26, p = 0.08; FR effect: F(2,70) = 43.48, p < 0.0001; interaction: p = 0.68]. These data provide some limited evidence that constitutive *Homer2* deletion increases demand for a low-concentration MA solution.

The dose-response function (5–40 mg/L MA) for active hole pokes under the original FR1 schedule of reinforcement was shifted upward in KO mice, compared to WT mice [Genotype effect: F(1,35) = 5.57, p = 0.02; interaction, p > 0.10], an effect especially apparent at lower MA doses (**Figure 4F**). KO mice also tended to exhibit higher active hole responding for the 80 mg/L solution (t-test, p = 0.09). Inactive hole pokes declined as a function of MA concentration, but no genotypic differences were detected (data not shown) [5–40 mg/L MA: Dose effect: F(2,70) = 4.19, p = 0.02; Genotype effect and interactions, p’s > 0.40; 80 mg/L: t-test, p = 0.25]. KO mice continued to show modestly higher relative responding on the active versus inactive lever during dose-response testing, but genotypic differences were not statistically significant (**Figure 4H**; 5–40 mg/L: Genotype X FR ANOVA, p’ > 0.10; at 80 mg/L, WT vs. KO: t-test, p = 0.06). Finally, in contrast to Homer2 knockdown (**Figure 2I**), the MA dose-intake function was shifted upwards in KO versus WT mice (**Figure 4I**) [5–40 mg/L: Genotype effect: F(1,35) = 4.70, p = 0.04; Dose effect: F(2,70) = 50.73, p < 0.0001; Genotype X Dose: p = 0.17; 80 mg/L: t(22) = 2.16, p = 0.04]. These latter data indicate that the potentiation of MA reinforcement and intake by constitutive *Homer2* deletion extends across a relatively broad dose-range.

### *Homer2b* Overexpression in the NAC Core, But Not Shell, Augments a MA-Induced CPP

**Figure 5:**
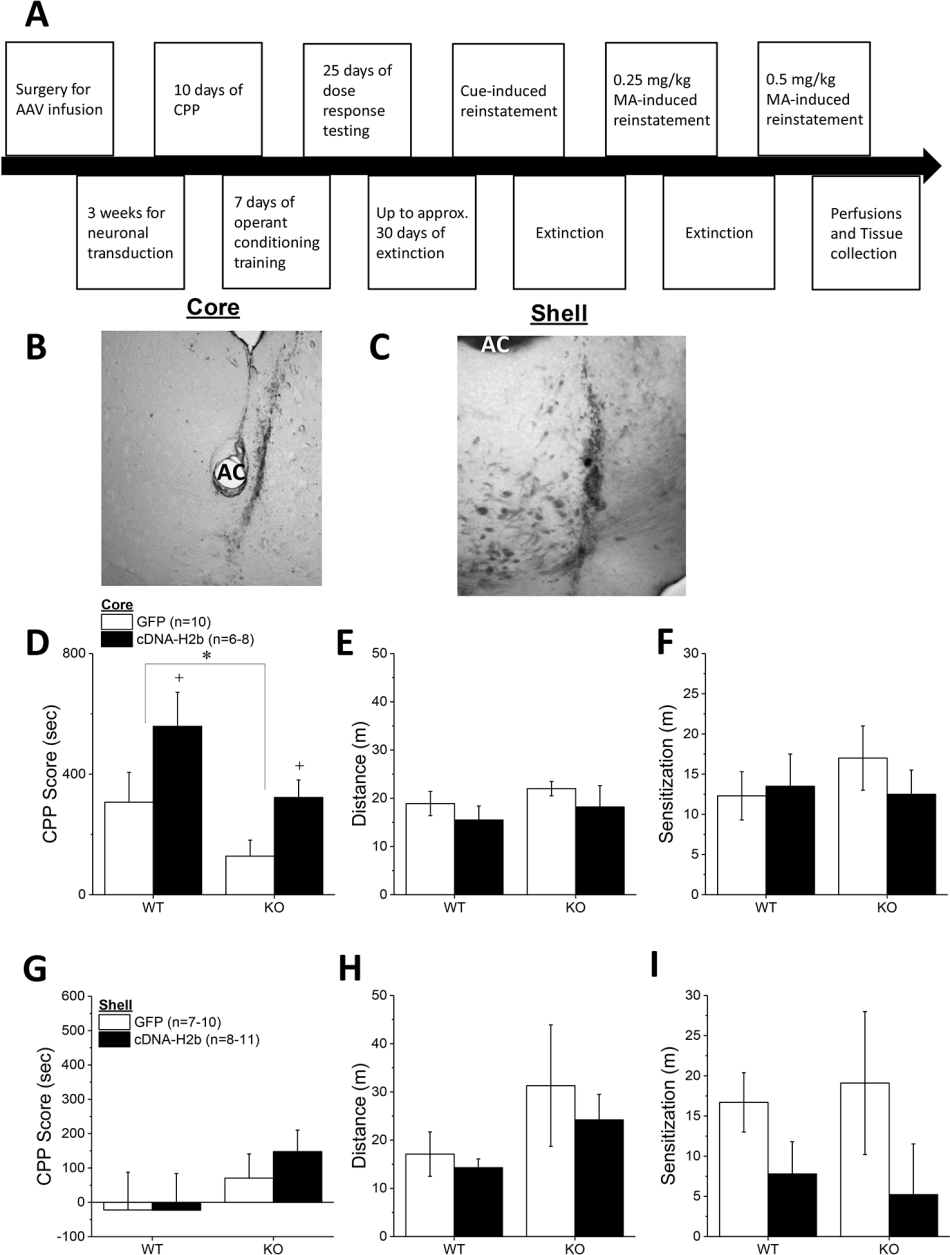
Homer2b overexpression in the nucleus accumbens (NAC) core, but not NAC shell, potentiates a methamphetamine (MA)-induced conditioned place-preference (CPP). **(A)** The procedural time-line for the study examining the effects of cDNA-mediated overexpression of Homer2b within the NAC core and shell. Representative micrographs of the neuronal transduction within the NAC core **(B)** and NAC shell **(C)** by antihemagglutinin (HA)-tagged adeno-associated viral vector (AAV)-cDNA encoding Homer2b (images at 20 X magnification). AC, anterior commissure. **(D)** The magnitude of a MA-induced CPP was lower in Homer2 knockout (KO) mice versus wild-type (WT) controls and cDNA infusion into the NAC core potentiated MA-induced place conditioning in both genotypes, without affecting the **(E)** acute or **(F)** sensitized locomotor response to MA. No genotypic difference or cDNA effect were apparent for **(G)** MA-induced CPP, **(H)** the acute locomotor response to MA or **(I)** the magnitude of MA-induced locomotor sensitization, when the cDNA was infused into the NAC shell. The data represent the means ± SEMs of the number of mice indicated in Panel **C** for NAC core and Panel **F** for NAC shell. ^*^p < 0.05 vs. WT; ^+^p < 0.05 vs. GFP.

The final series of experiments examined the effects of Homer2b overexpression within the NAC core and shell of *Homer2* WT and KO mice upon MA-induced place- and operant-conditioning. Immunohistochemical staining for the HA-tag indicated neuronal transduction within the NAC core (**Figure 5B**) that was comparable to that observed in prior reports from our group (e.g., 25, 28). Intriguingly, similar to NAC core knockdown of Homer2b (**Figure 1C**) and constitutive *Homer2* deletion (**Figure 3A**), Homer2b overexpression within the NAC core also potentiated the magnitude of a CPP induced by the repeated pairing of 2 mg/kg MA (**Figure 5D**) [AAV effect: F(1,33) = 7.18, p = 0.01]. While the initial dose-response study failed to support genotypic differences in the magnitude of the conditioned response elicited by pairing with 2 mg/kg MA (**Figure 3A**), the CPP elicited by this dose in the cDNA study was lower overall in KO versus WT mice (**Figure 5D**) [Genotype effect: F(1,33) = 6.20, p = 0.02; interaction: p = 0.74]. Homer2b overexpression within the NAC core did not influence the acute locomotor-response to 2 mg/kg MA (**Figure 5E**; Genotype X AAV ANOVA, p’s > 045) nor did it influence the sensitization of this response during conditioning (**Figure 5F**; Genotype X AAV ANOVA, p’s > 0.20). Thus, curiously, overexpressing Homer2b within the NAC core produces an effect on MA-conditioned reward akin to that observed upon either constitutive gene deletion (**Figure 3A**) or protein knockdown within this region (**Figure 1C**).

Immunohistochemical staining indicated robust neuronal transfection within the NAC shell, with no overt signs of infection or tissue damage (**Figure 5C**). Thus, we were surprised that the level of MA-induced place-conditioning was lower overall in the mice infused with GFP/cDNA into the NAC shell (**Figure 5G**), than that observed for the other place-conditioning experiments in this report. This low level of conditioning may have precluded our ability to detect group differences in the MA-conditioned response (Genotype X AAV, all p’s > 0.15). Despite lower CPP Scores, the locomotor response to an acute injection of 2 mg/kg MA was comparable to that observed in the other studies herein and was not affected by either *Homer2* deletion or intra-shell cDNA infusion (**Figure 5H**; Genotype X AAV ANOVA, all p’s > 0.07). Although intra-NAC shell cDNA infusion appeared to augment the difference in MA-induced locomotor activity observed from the first to the forth conditioning session (sensitization), this effect was not statistically significant (**Figure 5I**; Genotype X AAV ANOVA, all p’s > 0.06). These data do not support an active role for Homer2b within the NAC shell in gating MA-induced locomotion or -conditioned reward under place-conditioning procedures.

### *Homer2b* Overexpression Within NAC Subregions Does Not Influence the Acquisition of Oral MA Self-Administration

**Figure 6:**
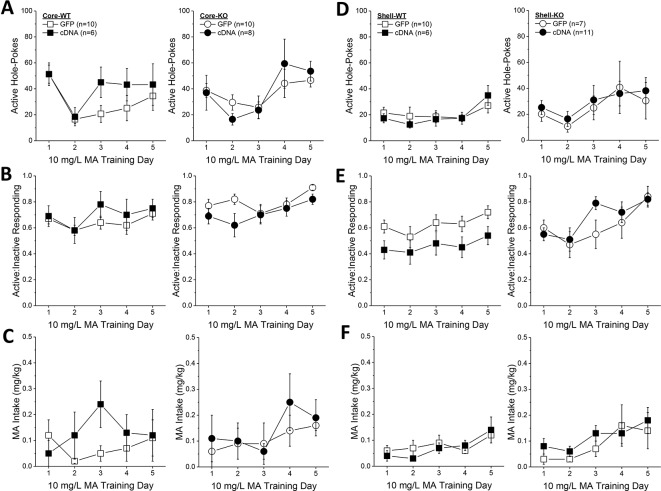
Homer2b overexpression does not alter methamphetamine (MA) reinforcement and intake during early training for MA self-administration. When compared to WT (left) and Homer2 knockout (KO) (right) mice infused with green fluorescent protein (GFP), cDNA infusion into the nucleus accumbens (NAC) core did not alter: **(A)** active hole responding, **(B)** response allocation or **(C)** MA intake during the first 5 days of self-administration training. **(D–F)** Similarly, cDNA infusion into the NAC shell did not affect any measure of self-administration during early training. The data represent the means ± SEMs of the number of mice indicated in Panel **A** (NAC core) and Panel **D** (NAC shell).

Based on the above results, we predicted that the effects of Homer2b overexpression upon place-conditioning would translate to operant-conditioning procedures. However, we found that Homer2b-cDNA infusion into either the NAC core (**Figures 6A–C**) or shell (**Figures 6D–F**) had no significant effect on any measure during the first 5 days of training under operant-conditioning procedures. A significant Genotype X Day interaction was detected for active hole responding in mice infused intra-NAC core (**Figure 6A**) [F(4,120) = 2.83, p = 0.03] that reflected a differential time-course of acquisition between WT and KO mice as *post hoc* comparisons between WT and KO mice failed to indicate genotypic differences in responding on any training day (t-tests, p’s > 0.15). In neither genotype did NAC core Homer2b overexpression alter active hole responding during the first 5 days of self-administration training (AAV effect and interactions, p’s > 0.20). In mice infused intra-NAC core, response allocation increased progressively during early training (**Figure 6B**) [Day effect: F(4,120) = 4.2, p = 0.003] and KO mice exhibited overall greater MA-appropriate responding than WT mice during this phase of study [Genotype effect: F(1,30) = 4.34, p = 0.05]. However, this measure was not altered by Homer2b overexpression within the NAC core (no AAV effect or interactions, p’s > 0.10). Finally, KO mice tended to consume more MA during the first 5 days of training (**Figure 6C**; Genotype X Day: p = 0.09), but there was no effect of intra-NAC core infusion of Homer2b cDNA upon MA intake during early training (AAV effect and interactions, p’s > 0.25). Taken together, these data do not support an effect of Homer2b overexpression within the NAC core in regulating initial MA reinforcement or intake.

Initial active hole responding was lower in mice infused with Homer2b-cDNA into the NAC shell than that typically observed under our oral MA operant-conditioning procedures (**Figure 6D**) and the Genotype X Day interaction failed to reach statistical significance [Day effect: F(4,128) = 4.79, p = 0.001; Genotype X Day, p = 0.095]. However, as observed for the NAC core, intra-NAC shell cDNA infusion did not alter active hole responding (AAV effect and interactions, p’s > 0.22). In mice infused intra-NAC shell, response allocation progressively increased across day, irrespective of the genotype or AAV treatment (**Figure 6E**) [Day effect: F(4,128) = 8.80, p < 0.0001; no interactions with the Day factor, p’s > 0.25]. However, in contrast to the data for the NAC core (**Figure 6B**), a significant Genotype X AAV interaction was detected for the ratio of active to inactive hole pokes exhibited by mice infused with AAV into the NAC shell (**Figure 6E**) [Genotype X AAV: F(1,32) = 5.0, p = 0.03]. Averaging across the 5 training days, this interaction reflected a cDNA-induced reduction in the response ratio in WT mice [t(16) = 3.29, p = 0.005], but no effect in KO animals (t-test, p = 0.83). Finally, MA intake fluctuated during early training in the mice infused intra-NAC shell (**Figure 6F**) [Day effect: F(4,128) = 8.08, p < 0.0001]. However, we detected no effect of gene deletion or intra-NAC shell cDNA infusion during the early training period (Genotype X AAV X Dose ANOVA, other p’s > 0.25).

### *Homer2b* Overexpression Within the NAC Shell Reduces the Efficacy of Oral MA to Serve as a Positive Reinforcer

**Figure 7:**
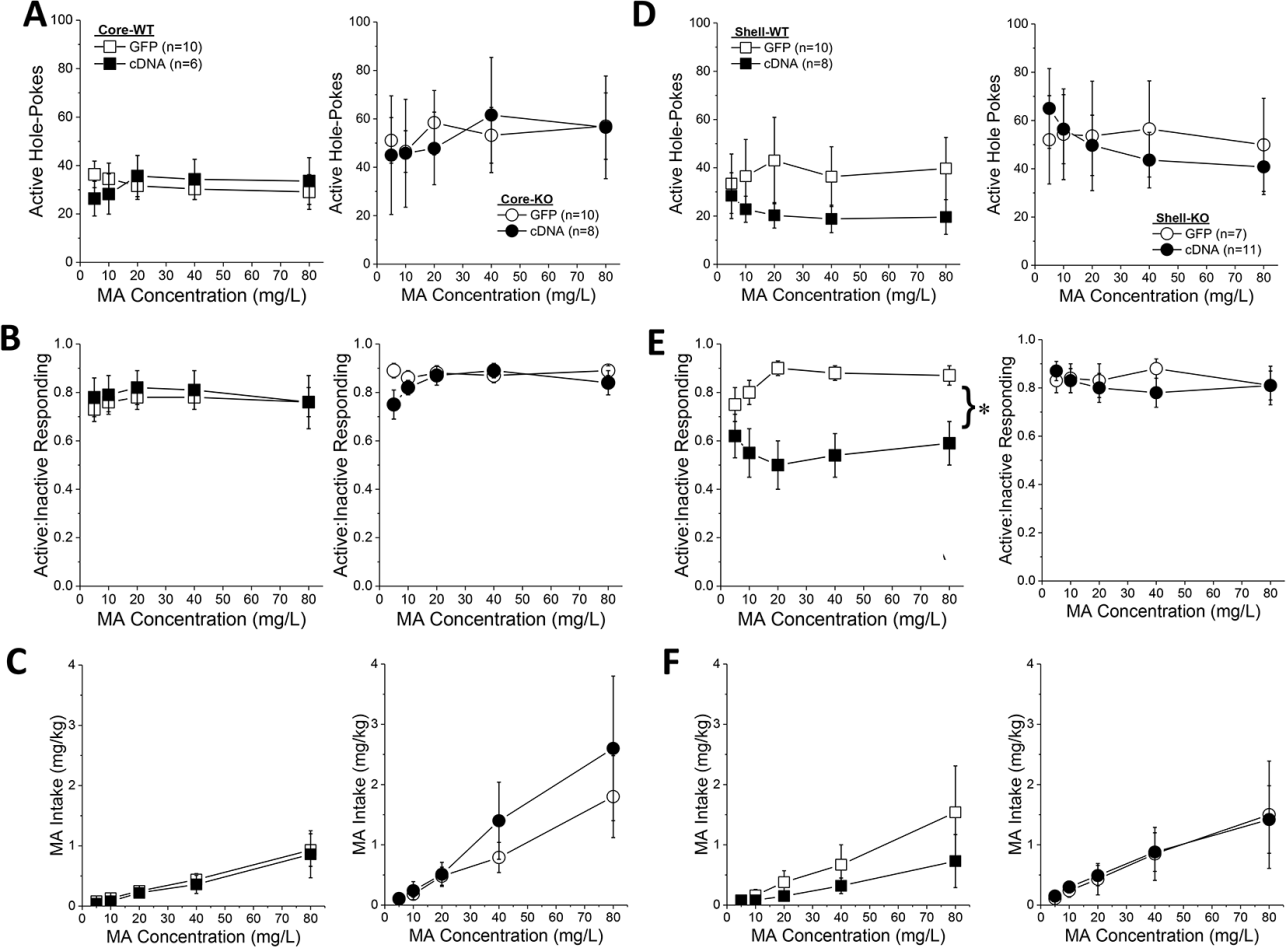
Homer2b overexpression in the nucleus accumbens (NAC) shell blunts methamphetamine (MA) reinforcement only in wild-type (WT) mice. When compared to WT (left) and Homer2 knockout (KO) (right) mice infused with green fluorescent protein (GFP), cDNA infusion into the NAC core did not alter the dose-response functions for: **(A)** active hole responding, **(B)** response allocation or **(C)** MA intake. **(D)** cDNA infusion into the NAC shell caused a declining dose-response function for active hole-responding in both WT and KO mice, **(E)** lowered the dose-response function for response allocation in the active hole in WT mice, but **(F)** did not significantly alter the dose-response function for MA intake. The data represent the means ± SEMs of the number of mice indicated in Panel A (NAC core) and Panel **D** (NAC shell). ^*^p < 0.05, main AAV effect. Main Genotype effects are not indicated for clarity but are described in the text.

Homer2b-cDNA infusion into the NAC core (**Figures 7A–C**) did not influence any self-administration measure as a function of the concentration of the MA reinforcer. The dose-response function for active hole-poking was relatively flat in mice infused intra-NAC core with our AAVs and there was no effect of *Homer2* deletion or AAV infusion upon this measure (**Figure 7A**; Genotype X Dose X AAV ANOVA, all p’s > 0.12). Although KO mice tended to exhibit a higher ratio of active versus inactive responding during dose-response testing (Genotype effect, p = 0.07), no significant group differences were detected for this measure at any MA dose tested (**Figure 7B**; Genotype X Dose X AAV ANOVA, other p’s > 0.15). In this experiment, *Homer2* deletion shifted the dose-response for MA intake (**Figure 7C**) [Genotype X Dose: F(4,120) = 3.04, p = 0.02], but *post hoc* tests failed to confirm genotypic differences at any MA dose (t-tests, p’s > 0.07) and no AAV effects or interactions were detected (p’s > 0.40). Taken together, these cDNA data argue against an active role for Homer2b within the NAC core in regulating MA intake or sensitivity to its reinforcing effects.

In contrast to the NAC core, Homer2b-cDNA infusion into the NAC shell altered the dose-response function for active nose-poking behavior (**Figure 7D**) [AAV X Dose: F(4,120) = 2.89, p = 0.03]. Although inspection of **Figure 7D** suggested that this interaction was driven by the results from the WT mice, there was no genotype effect or interactions with the genotype factor (Genotype effect: p = 0.10; all interactions with Genotype factor: p’s > 0.40). Collapsing the data across genotype, *post hoc* analyses did not indicate any significant GFP-cDNA difference at any of the MA concentrations tested (p’s > 0.25), arguing that the AAV X Dose interaction reflected the distinct shapes of the dose-response functions for GFP- versus cDNA-infused mice (respectively, flat vs. descending). Homer2b-cDNA into the NAC shell also altered the dose-response function for the ratio of active versus inactive responding (**Figure 7E**) [AAV X Dose: F(4,120) = 2.89, p = 0.03] - an effect driven by the shift down-wards in the dose-response response produced by cDNA infusion in the WT mice (**Figure 7E**) [Genotype X AAV: F(1,30) = 4.20, p = 0.05]. While it appeared that an intra-NAC shell infusion of Homer2b-cDNA lowered MA intake selectively in WT mice (**Figure 7F**), no group differences were observed with respect to the MA dose-intake function [Dose effect: F(1,124) = 12.73, p < 0.0001; all other p’s > 0.40]. Taken together, these data argue that Homer2b overexpression within the NAC shell lowers the efficacy of MA to serve as a reinforcer, without significantly impacting MA intake.

### *Homer2b* Overexpression Within NAC Subregions Does Not Alter the Extinction or Reinstatement of MA-Seeking

**Figure 8:**
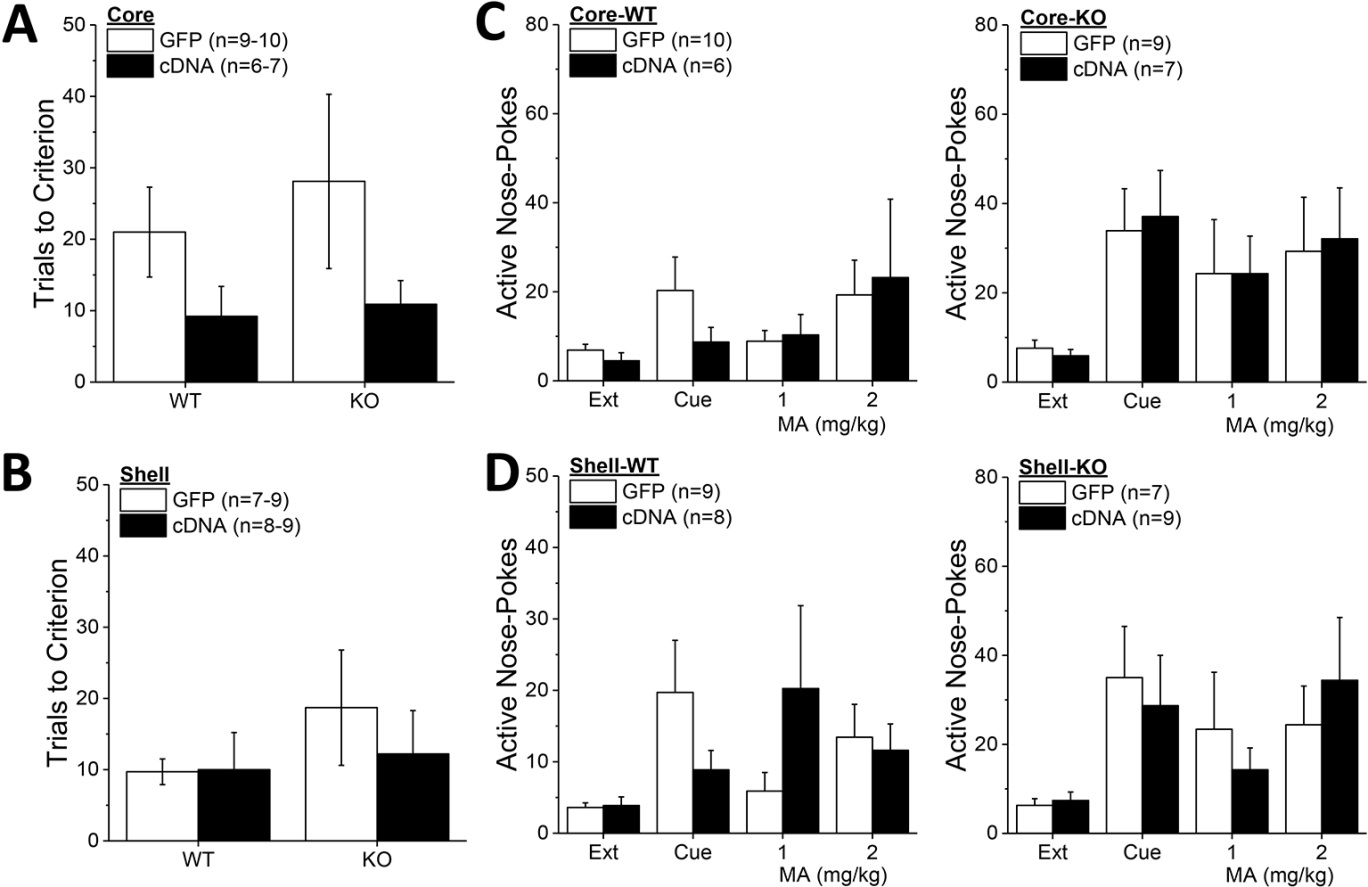
Homer2b overexpression in nucleus accumbens (NAC) subregions does not significantly alter genotypic differences in responding under extinction-reinstatement procedures. Although it appeared that cDNA infusion into the NAC core reduced the number of trials taken to reach extinction criterion in both wild-type (WT) and Homer2 knockout (KO) mice, no cDNA effect was observed on this measure when infused into either the NAC core **(A)** or the NAC shell **(B)**. **(C)** Homer2 KO mice exhibited greater cue- and MA-primed reinstatement of active hole responding than WT mice, but NAC core infusion of cDNA did not affect responding in either WT (0left) or KO animals (right). **(D)** cDNA infusion into the NAC shell also did not alter the genotypic difference in reinstatement. The data represent the means ± SEMs of the number of mice indicated in their respective panels. Main Genotype effects are not indicated for clarity but are described in the text. The sample sizes in this figure are lower than those indicated in **Figures 6** and **7** as some animals were euthanized following MA self-administration procedures due to illness.

Although Homer2b-cDNA infusion into the NAC core appeared to reduce the number of trials to reach extinction criterion in both WT and KO mice (**Figure 8A**), no group differences were detected for this measure (Genotype X AAV ANOVA, p’s > 0.09). Likewise, neither *Homer2* deletion nor Homer2b-cDNA infusion into the NAC shell altered the time taken to extinguish responding in the active hole (**Figure 8B**; Genotype X AAV ANOVA, p’s > 0.31). A comparison of active hole responding during the last day of extinction with that elicited by presentation of the MA-associated cue or a priming injection of 1 or 2 mg/kg MA indicated greater responding, overall, in *Homer2* KO versus WT mice, irrespective of the AAV infused into the NAC core (**Figure 8C**) [Test effect: F(3,81) = 6.31, p = 0.001; Genotype effect: F(1,27) = 5,26, p = 0.03; no AAV effect and no interactions, p’s > 0.25]. Inspection of **Figure 8C** suggested that cDNA into the NAC core differentially affected the magnitude of cue-induced reinstatement (0 mg/kg MA), while exerting no effect on MA-primed responding. However, a direct comparison of responding on the cued reinstatement test and the extinction baseline failed to detect any interaction with the AAV factor [AAV effect and interactions, p’s > 0.30; Genotype X Test: F(1,28) = 5.91, p = 0.02]. Akin to the findings for the NAC core, cDNA infusion into the NAC shell also did not significantly influence active hole responding during the tests for reinstatement of drug-seeking (**Figure 8D**; no AAV effect or interactions, p’s > 0.17), but again, *Homer2* KO mice exhibited greater responding overall, compared to WT mice [Genotype effect: F(1,27) = 4.40, p = 0.04]. These data for the extinction and reinstatement of MA-seeking argue a suppressive role for Homer2 in regulating behavior but do not support either NAC subregion as the active loci of these effects.

## Discussion

Homer2 is a postsynaptic scaffolding protein regulating the localization and function of glutamate receptors [c.f., (37–44)] and its expression within the NAC plays a necessary and active role in behavioral sensitivity to both cocaine and alcohol [c.f., (27, 37)]. In more recent work (8), idiopathic, genetic, and MA-induced vulnerability to MA addiction-related behaviors was found to be associated with increased indices of glutamate signaling within the NAC, including elevated Homer2 expression. More specifically, the magnitude of a MA-induced CPP is highly correlated with Homer2 expression within both the shell and core subregions of the NAC, arguing a potential role for Homer2-dependent neuroadaptations within both subregions in the motivational valence of MA. However, in both genetically vulnerable MAHDR and MA-sensitized B6 mice, increased behavioral sensitivity to MA was associated with elevated Homer2 expression within the NAC shell only, suggesting some subregional specificity may exist within the NAC regarding the relationship between Homer2-dependent signaling and MA-induced behaviors. Using an shRNA strategy to selectively knockdown the major rodent isoform of Homer2 [Homer2b; (45)] within the NAC shell, we demonstrated previously little to no effect upon the magnitude of a MA-CPP, but a marked reduction in responding for oral MA reinforcement and for MA intake when the same mice were assayed under operant-conditioning procedures. Such data argued a necessary role for Homer2b within the NAC shell for MA reinforcement/intake and suggested that idiopathic or MA-induced increases in NAC shell Homer2b expression promotes a MA addicted phenotype (8). Herein, we extended the results of this prior study to the NAC core.

Further, to determine whether Homer2b within either NAC subregion actively regulates MA-induced changes in behavior, we also applied our cDNA-Homer2b strategy [e.g., (25, 26, 28, 34)] in both WT and constitutive *Homer2* KO mice to upregulate Homer2b expression. The results show that constitutive *Homer2* deletion potentiated MA-CPP, oral MA reinforcement/intake under operant-conditioning procedures, and the reinstatement of MA-seeking following response extinction. A subset of these KO effects was recapitulated by Homer2b knockdown in the NAC core, providing new evidence that Homer2b within the NAC shell and core oppositely regulate MA reinforcement. While the present shRNA findings argue a suppressive role for NAC core Homer2b expression in regulating MA reward/reinforcement, cDNA-Homer2b infusion within this subregion also potentiated a MA-CPP, without affecting measures of MA reinforcement/intake/reinstatement. Opposite our expectations (8), Homer2b overexpression within the NAC shell reduced indices of MA reinforcement but did not affect other behavioral measures. Below, we discuss these effects of bidirectional manipulations of Homer2b within the NAC in the context of animal models of MA reward and reinforcement. The data collected during this study are summarized in **Table 1**.

**Table 1:** Summary of the results of the present experiments.

Behavioral Measure	Core Knockdown	Constitutive KO	Core Over-expression: Effects of KO	Core Over-expression: Effects of cDNA	Shell Over-expression: Effect of KO	Shell Over-expression: Effect of cDNA
CPP	↑	↑	↓	↑	–	–
Locomotor Sensitization	–	--	–	–	–	–
Self-Administration Training	–	↑	–	–	--	–
Increasing Response Requirement	–	↑	N/D	N/D	N/D	N/D
Self-Administration Dose Response Curve	↑	↑	–	--	–	↓
Trials to Extinction	N/D	N/D	–	–	--	–
Cue- & MA-induced reinstatement of self-administration	N/D	N/D	↑	–	–	

↑ denotes an increase in behavior relative to control. ↓ denotes a decrease in behavior relative to control. – denotes no effect of manipulation relative to control. N/D denotes not determined.

### Constitutive Homer2 Deletion Tends to Promote MA Reward/Reinforcement

Constitutive *Homer2* deletion potentiated: MA-conditioned reward (**Figure 3A**), responding for oral MA reinforcement and MA intake under operant-conditioning procedures (**Figures 4** and **7**), and the number of trials required to extinguish MA-seeking behavior (**Figure 8A**). Additionally, *Homer2* deletion increased the magnitude of both cue- and MA-primed reinstatement of MA-seeking behavior following extinction (**Figures 8C, D**). Further, the increased MA reinforcement and intake observed in *Homer2* KO mice was apparent early during self-administration training (**Figures 4A–C**) and persisted across a range of MA doses in MA-experienced mice (**Figures 4G–I**). Such data argue a suppressive role for Homer2 in both gating vulnerability to early MA abuse and maintaining an addicted phenotype. Although genotypic differences in MA-induced locomotor activity were not observed in the present study (**Figures 3B, C**), we reported previously that the dose-response function for acute MA-induced locomotor activity is shifted upwards in *Homer2* KO mice versus WT controls (36). Thus, it is possible that the increased MA reinforcement and intake exhibited by *Homer2* KO mice herein relates to the greater efficacy of the drug to induce psychomotor activation. The precise reason for the present failure to replicate genotypic differences in MA-induced locomotion is not entirely clear but likely reflects procedural differences between the studies. First and foremost, the two studies were conducted in two distinct research institutions (Medical University of South Carolina vs. University of California Santa Barbara); thus a host of environmental differences may have contributed to the differential results to include the fact that the mice in the present study were bred in-house, while those in our earlier study were obtained from the laboratory of Dr. P.F. Worley at Johns Hopkins University School of Medicine. Also, the present experiments employed both a shorter testing period (15 vs. 60 min) and a smaller testing arena than our prior report. Additionally, mice in the present study underwent saline-conditioning sessions on the days intervening between MA injections, while mice in the prior study were injected with MA only (36). Nevertheless, the present data for MA reward/reinforcement in *Homer2* KO mice aligns well with those reported for cocaine reward/reinforcement (25), providing new evidence for a generalization of a “proaddictive” phenotype of *Homer2* KO mice across different psychomotor stimulant drugs of potential relevance for the neurobiology of psychomotor stimulant abuse liability and/or MA-cocaine coabuse.

Drawbacks of a constitutive KO approach for studying the neurobiology of behavior relate to the lack of developmental and neuroanatomical specificity of gene deletion. There exist three different Homer isoforms, with Homer1 and Homer2 isoforms expressed in midbrain and forebrain regions highly implicated in addiction neurobiology (45). Further, distinct Homer1 isoforms differentially regulate spontaneous and stimulant-induced changes in behavior (32, 36), with imbalances in the relative expression of Homer1 versus Homer2 isoforms within mPFC gating cocaine-conditioned reward (34) and the reinstatement of cocaine-seeking behavior (46). Although extant correlative evidence does not support a relationship between Homer1 protein expression within either NAC subregion or within the mPFC and MA behavioral sensitivity (4, 8), such findings do not preclude the possibility that compensatory changes in Homer1 expression/function may contribute to the “proaddictive” phenotype of *Homer2* KO mice.

### Subregional Selectivity in the Effects of *Homer2b* Knock-Down Within NAC Upon MA Reward/Reinforcement

In brain, Homer2 expression is regulated in a regionally selective manner by prior MA experience in inbred B6 mice, with increases in protein expression observed selectively within the NAC shell (4, 8). Further, increased Homer2 expression within the NAC shell, but not core, is a biochemical correlate of genetic vulnerability to consume MA in mice on a heterogeneous genetic background (8). Providing causal evidence that Homer2 functions to alter MA reward/reinforcement in a subregionally distinct manner, Homer2b knockdown in the shell reduces (8), while knockdown in the core increases, both the magnitude of a MA-CPP and responding for a MA reinforcer (**Figures 1** and **2**). Thus, the effects of constitutive *Homer2* deletion upon MA reward/reinforcement/intake (**Figures 3** and **4**) are recapitulated, albeit incompletely, by Homer2b knockdown within the NAC core (**Figures 1** and **2**; **Table 1**). Given the neuroanatomical nature of our research question, we deemed it more critical to decipher the site of AAV transduction within NAC subregions than quantify the efficiency of our shRNA construct to alter Homer2b protein expression. The fact that the phenotype produced by Homer2b knockdown in the NAC core did not fully recapitulate that of the *Homer2* KO mouse is perhaps not surprising as we know from prior work that our shRNA-Homer2b infusion procedure consistently reduces protein expression by 40%–50% *in vivo* (31, 33, 34, 47) and does not completely eliminate protein expression as is the case for gene deletion. Further, in humans, MA addiction is associated with anomalies in the function of many brain structures that were not targeted herein (48–50). Indeed, lower Homer2 expression within the mPFC of mice is associated with both genetic and idiopathic vulnerability to express a MA-CPP, and to respond for/consume the drug under operant-conditioning procedures (4). Thus, it is highly likely that Homer2 within other structures embedded within putative addiction neurocircuits functions also to regulate MA-conditioning, MA-seeking and MA-taking behavior and contribute to the robust MA phenotype of *Homer2* KO mice. Although Homer2b knock-down in the NAC core does not fully recapitulate the effect of constitutive gene deletion, it is interesting to note that the phenotype produced by Homer2b knock-down in the NAC core predominates in the *Homer2* KO mouse (**Table 1**).

In our limited experience using shRNA to target Homer2 expression within both NAC subregions (29) and to the best of our knowledge of the extant Homer2 literature, our shRNA-Homer2 findings for MA reward/reinforcement [(8); present study] are the first to demonstrate opposing roles for Homer2b within NAC subregions in regulating addiction-related behavior. We know through studies of constitutive *Homer2* KO mice and of the effects of intracranial shRNA-Homer2b infusion that intact Homer2 expression is important for: maintaining basal extracellular glutamate levels within both the NAC and mPFC (25, 34, 47), cocaine- and alcohol-stimulated glutamate release (25, 26, 32, 33), and the expression/function of glutamate receptors, transporters, and signaling molecules within these regions (25, 26, 34). However, we are unaware of any study that has directly compared the effects of either *Homer2* deletion or Homer2b knockdown upon any biochemical measure *between* NAC subregions to inform the mechanisms underpinning the opposing MA effects of Homer2b knockdown observed herein. That being said, we do know from studies of the mPFC that shRNA-Homer2b (and cDNA-Homer2b) infusion can produce not only local effects upon basal and drug-stimulated changes in extracellular glutamate, in addition to changes in the expression of Homer2 and glutamate receptor-related proteins, but can also alter these biochemical measures within NAC, intriguingly in a direction sometimes *opposite* that observed at the site of infusion (34). As striking examples, intra-mPFC infusion of cDNA-Homer2b elevates basal extracellular glutamate content and Homer2 expression, in addition to blunting drug-stimulated glutamate release at the site of infusion, but lowers the glutamate content and expression of both Homer1/2 and mGlu1/5 and potentiates drug-stimulated glutamate release within the NAC. Further, intra-mPFC infusion of shRNA-Homer2b reduces Homer2 and mGlu5 expression at the infusion site, but elevates markedly the expression of GluN2b, without affecting extracellular glutamate or the expression of Homers or mGlu1/5 within the NAC (34). These adaptations within NAC cannot be readily explained by anterograde transport of the AAVs and argue that the opposing effects of shRNA-Homer2b infusion into the NAC shell and core observed herein could reflect yet uncharacterized distinctions in local changes in extracellular glutamate and/or glutamate receptor function/expression that differentially alter the activation of efferents or could reflect yet uncharacterized biochemical alterations within those efferent structures (e.g., ventral pallidum).

### Inconsistent Effects of Increasing *Homer2b* Expression Within the NAC Shell and Core Upon MA Reward/Reinforcement

The observed effects of intra-NAC core/shell shRNA-Homer2b infusion argued that Homer2b expression within the NAC core suppresses, while that in the NAC shell promotes, certain MA addiction-related behaviors in mice [**Figures 1** and **2**; (8)]. However, when this hypothesis was tested directly using well-established AAV-cDNA approaches that increase local Homer2b expression by approximately 50% (25, 26, 28, 34, 47), we found no supporting evidence for either notion. If anything, the results from our cDNA study were opposite those predicted from our shRNA experiments. For one, intra-NAC shell infusion of cDNA-Homer2b lowered the dose-response functions for MA-reinforced/appropriate responding in WT mice (**Figures 5F**, **6D, E**)—an effect qualitatively similar to (albeit more robust than) that observed upon Homer2b knockdown in this subregion of B6 animals (8). Also, an intra-NAC core infusion of either shRNA-Homer2b in B6 mice or cDNA-Homer2b in B6-129 hybrid WT mice produced a quantitatively similar increase in the magnitude of a MA-CPP (**Figure 1D** vs. **Figure 5E**). Despite baseline differences in responding, Homer2b overexpression and underexpression within NAC core produces similar effects upon the MA-conditioned reward expressed in each experiment. Finally, within the context of operant-conditioning, in no instance did cDNA-Homer2 infusion into either NAC subregion significantly alter, let alone reverse, the MA phenotype of *Homer2* KO mice (**Figures 7** and **8**).

These null data are in stark contrast to our earlier reports demonstrating a complete reversal of the behavioral and/or neurochemical phenotype of *Homer2* KO mice by site-directed infusions of our cDNA-Homer2b construct (25, 26, 34). In only one instance did the data for cDNA-Homer2b infusion align with our predictions and this was observed within the context of extinction/reinstatement procedures. Although the results failed to reach statistical significance, cDNA-Homer2b infusion into the NAC core facilitated the extinction of operant-behavior (an effect observed in both WT and KO mice; **Figure 8A**) and blunted the capacity of the MA-associated cues to reinstate responding in WT mice (**Figure 8B**). That being said, cDNA-Homer2b infusion into the NAC shell produced a comparable reduction of cue-induced reinstatement as that observed in mice infused intra-NAC core (**Figure 8A** vs. **Figure 8B**). Such null results argue strongly against an active and autonomous role for Homer2 within either NAC subregion in regulating MA-conditioned reward or self-administration. Alternatively, these data could also suggest that any dysregulation in Homer2 expression, be it overexpression or underexpression, is sufficient to perturb normal glutamate transmission within NAC subregions to affect MA reward/reinforcement. Which, and how, specific signal transduction pathways are affected by increasing versus decreasing Homer2 expression within different NAC subregions remains to be determined and are important research questions for future studies aimed at understanding more precisely the role played by this scaffolding protein in regulating MA addiction-related behaviors.

### Additional Caveats of the Current Study

**Table 1** summarizes the major findings from this study, which are complicated to interpret to say the least. Adding to the interpretational difficulty is the notable fact that the baseline behavior of the control animals varied considerable across the different experiments. For instance, the baseline CPP behavior of GFP-infused B6 mice in the shRNA study of the NAC core was approximately half that of the WT B6-129 mice in the cDNA study of this region (**Figure 1C** vs. **Figure 5D**). These experiments were conducted over a year apart; thus, we cannot decipher from the current experimental design whether or not this difference in baseline CPP reflects environmental factors (e.g., differences in laboratory or animal care personnel) or strain differences in behavioral sensitivity to MA. Indeed, marked strain differences are reported between C57BL/6J mice and DBA2/J mice with respect to MA intake, with C57BL/6J mice exhibiting significantly lower MA intake than DBA2/J mice [e.g., (51–53)]. To date, we have yet to directly compare MA CPP, reinforcement or intake between B6 mice and mice on a mixed B6-129 background so we cannot rule out the potential contribution of background strain to our findings. However, arguing more in favor of environmental factors as contributors to the differences in baseline CPP, the 2 mg/kg MA dose elicited negligible CPP in the B6-129 mice infused with cDNA into the NAC shell (**Figure 5G**), despite these animals exhibiting similar acute and sensitized locomotor responses to the drug as those infused with cDNA into the NAC core (**Figures 5E, F** vs. **Figures 5H, I**). The MA self-administration behavior of the B6-129 mice infused with cDNA into the NAC shell was also lower than that exhibited by their NAC core counterparts, particularly during the training phase of the experiment (**Figure 6**). Such behavioral differences cannot be attributable to differences in genetic background.

Further, we would like to be forthcoming and report that, unfortunately, during the year we were conducting the NAC shell cDNA study, building renovations were occurring on the level beneath our laboratory. While arrangements were in place to minimize the noise and vibration during the daylight hours when the animals were being tested, we cannot rule out the possibility that the construction conducted during the evening hours affected the behavior of the animals nor did we have any control over, or ability to predict, any construction that took place during the day. For this very reason, the cDNA study of the NAC shell was conducted in 3 distinct cohorts of 21–25 B6-129 mice, spaced 1–3 months apart in accordance with the limited information we were provided regarding heavy construction/demolition. However, despite our best attempts to avoid this confound, we were unsuccessful at eliciting a CPP in this experiment. Indeed, the number of mice exhibiting a conditioned place-aversion [CPP Score <–100 s; see (8)] in each cohort of the cDNA study of the NAC shell was higher than that observed in the cDNA study of the NAC core (shell: 3–5/cohort vs. core: 2–3/cohort), with more mice exhibiting place-ambivalence. It is also possible that the AAV-GFP infusion into the NAC shell might have inadvertently affected the behavior of the B6-129 mice, although we observed no overt signs of infection or tissue damage. However, we deem this unlikely as we have conducted numerous experiments in which this AAV was infused into the NAC shell, to include studies of MA- (8), alcohol- (26, 28), and cocaine-induced place-conditioning (25) and observed no obvious off-target effects of the AAV upon the expression of the conditioned response. Thus, we surmise that factors related to building renovations likely confounded data interpretation from the cDNA study of the NAC shell.

### Concluding Remarks and Future Directions

Considerable neuropharmacological, chemogenetic, and optogenetic work has established that the NAC shell and core are embedded within distinct neural subcircuits that differentially contribute to aspects of drug-conditioning, drug-taking, and drug-seeking behavior, the most well characterized of which are the relatively dense afferents from, respectively, the infralimbic (IL) and prelimbic (PL) subregions of the mPFC [e.g., (54, 55)]. The majority of data argue that PL-NAC (core) projections are involved in driving or executing operant behavior in the context of drug self-administration, whereas IL-NAC (shell) projections are more critical for suppressing or inhibiting responding [e.g., (56, 57)]. This being said, there is overlap in the PL and IL projections to specific NAC subregions (55, 58) that can bear on how specific corticoaccumbens projections might influence responding for drugs and natural reinforcers [see (54, 59, 60)]. Thus, while the available data pertaining to Homer2 regulation of MA addiction-related behavior in mice do not reliably support an active role for Homer2 within NAC subregions for gating MA addiction-related behaviors, our AAV findings do not negate a role for this Homer isoform within NAC afferents, in particular those from the mPFC, in this regard. Although repeated MA does not alter Homer2 expression in samples from the entire PFC (to include PL, IL, and anterior cingulate), reduced PFC Homer2 expression is associated with both genetic and idiopathic MA addiction vulnerability in mouse models (4). Given the importance of mPFC-NAC subcircuits for gating drug-taking and drug-seeking behavior and based on our earlier cocaine studies of Homer2 function within mPFC (34), one goal of future work is to characterize the neuroanatomical selectivity of MA-induced changes in Homer2/glutamate signaling within PFC subregions and to interrogate the role played by distinct mPFC-NAC subcircuits and Homer2 expression within these subcircuits in MA-taking and MA-seeking behavior.

## Data Availability Statement

The datasets generated for this study are available on request to the corresponding author.

## Ethics Statement

The animal study was reviewed and approved by Institutional Care and Use Committee of the University of California Santa Barbara.

## Author Contributions

CNB, TK, CDB, and KS designed the experiments. CNB, JS, EF, AP, DF, SF, and EL composed initial drafts of experimental write-ups. CNB and KS conducted the data analyses, consolidated findings, and composed the final manuscript. CNB, JS, EF, AP, DF, EL, SR, and SF performed the experiments and GJ provided and consulted on the viruses. All co-authors edited the manuscript.

## Funding

This work was funded by NIH/NIDA grant DA039168 (CDB and KS).

## Conflict of Interest

The authors declare that the research was conducted in the absence of any commercial or financial relationships that could be construed as a potential conflict of interest.
